# ZnO/PVDF Nanogenerators with Hemisphere-Patterned PDMS for Enhanced Piezoelectric Performance

**DOI:** 10.3390/polym17152041

**Published:** 2025-07-26

**Authors:** Kibum Song, Keun-Young Shin

**Affiliations:** 1 Department of Convergence of Energy Policy and Technology, Soongsil University 369, Sangdo-ro, Dongjak-gu, Seoul 06978, Republic of Korea; kibumsong121@naver.com; 2 Department of Materials Science and Engineering, Soongsil University 369, Sangdo-ro, Dongjak-gu, Seoul 06978, Republic of Korea

**Keywords:** ZnO, PVDF, PDMS, electrospinning, nanogenerator

## Abstract

In this study, we present a flexible piezoelectric nanogenerator based on a zinc oxide (ZnO)/polyvinylidene fluoride (PVDF) nanocomposite electrospun onto a hemisphere-patterned PDMS substrate. The nanogenerator was fabricated by replicating a silicon mold with inverted hemispheres into PDMS, followed by direct electrospinning of ZnO-dispersed PVDF nanofibers. Varying the ZnO concentration from 0.6 to 1.4 wt% allowed us to evaluate its effect on structural, dielectric, and piezoelectric properties. The nanogenerator containing 0.8 wt% ZnO exhibited the thinnest fibers (371 nm), the highest β-phase fraction (85.6%), and the highest dielectric constant (35.8). As a result, it achieved the maximum output voltage of 7.30 V, with excellent signal consistency under an applied pressure of 5 N. Comparisons with pristine PVDF- and ZnO/PVDF-only devices demonstrated the synergistic effect of ZnO loading and patterned PDMS on the enhancement of piezoelectric output. The hemisphere-patterned PDMS substrate improved the mechanical strain distribution, interfacial contact, and charge collection efficiency. These results highlight the potential of ZnO/PVDF/PDMS hybrid nanogenerators for use in wearable electronics and self-powered sensor systems.

## 1. Introduction

Recent advancements in wearable electronics and energy-harvesting technologies have created a growing demand for flexible and self-powered sensors. These sensors can be broadly categorized based on their energy conversion mechanisms, including piezoelectric, triboelectric, ion gradient-based, and electrochemical sensors. Among various approaches, piezoelectric nanogenerators have attracted considerable attention due to their ability to convert mechanical deformation—such as body movement—into electrical signals or usable energy [1,2,3]. Their low power consumption, compact form factor, and mechanical flexibility make them ideal for wearable applications requiring continuous operation and user comfort. Furthermore, the development of self-powered systems significantly reduces reliance on external power sources and battery replacements, which is crucial for long-term health monitoring and human–machine interfaces [4,5,6].

Piezoelectric materials are central to these applications, and much research has focused on enhancing their performance and device integration [7]. Zinc oxide (ZnO) is a widely studied piezoelectric material known for its strong electromechanical coupling, wide bandgap, and high chemical stability [8]. Although ZnO nanostructures—such as nanorods and nanowires—exhibit excellent piezoelectric properties, their intrinsic brittleness limits their use in standalone devices. To address this, ZnO is often incorporated into flexible polymer matrices, particularly polyvinylidene fluoride (PVDF), forming composite structures that combine mechanical flexibility with enhanced piezoelectric output [9].

PVDF is a semi-crystalline polymer whose piezoelectric properties are primarily attributed to its β-phase content. While the α-phase exhibits randomly oriented dipoles, the β-phase aligns molecular dipoles along the polymer chain, resulting in higher polarization and dielectric constants [10,11]. Electrospinning is a widely used technique to fabricate PVDF nanofibers, as the high-voltage field promotes chain alignment and β-phase formation [12]. Incorporating ZnO nanoparticles during electrospinning serves dual functions: enhancing β-phase nucleation and improving charge generation under mechanical stress.

To further increase the mechanical stability and output performance of nanogenerators, polydimethylsiloxane (PDMS) is often employed as a flexible substrate. PDMS is biocompatible, mechanically robust, and can be easily patterned to create micro- and nanostructures that amplify surface stress during deformation [13,14]. While various patterning methods for PDMS such as soft lithography and plasma-induced wrinkling have been reported, hemisphere-patterned structures offer several advantages due to their isotropic geometry, including uniform strain distribution, reduced stress concentration, and improved interfacial contact and mechanical durability. When combined with piezoelectric materials, PDMS not only enhances flexibility and durability but also facilitates effective stress transfer, boosting energy conversion efficiency. Moreover, ZnO, PVDF, and PDMS are all non-toxic and biocompatible, making them well-suited for applications involving direct skin contact, such as wearable health monitors and bio-integrated energy devices [15,16].

In this study, we explore the synergistic effects of ZnO content, PVDF crystallinity, and PDMS surface morphology on the performance of composite piezoelectric nanogenerators. A ZnO/PVDF hybrid solution was electrospun onto a hemisphere-patterned PDMS substrate to fabricate a flexible nanogenerator. To date, few studies have systematically investigated the combined effect of optimized ZnO loading and hemispherical PDMS surface patterning on the piezoelectric performance of PVDF-based nanogenerators. This work addresses that gap by demonstrating their synergistic influence through detailed material characterization and device performance analysis. The structural and material properties were analyzed using scanning electron microscopy (SEM), Fourier-transform infrared spectroscopy (FT-IR), Raman spectroscopy, and X-ray diffraction (XRD). Finally, the piezoelectric output performance of nanogenerators with varying ZnO loadings was evaluated using an oscilloscope. This work aims to provide insights into the optimization of composite structures for next-generation self-powered wearable electronics.

## 2. Experimental Section

### 2.1. Materials

A positive photoresist (PR, GXR601) and developer (AZ 325) were purchased from AZ Electronic Materials. Polyvinylidene fluoride (PVDF, Kynar Flex^®^ 2801-00, Arkema (Colombes, France)), polydimethylsiloxane (PDMS, Sylgard 184, Dow Corning (Midland, MI, USA)), sulfuric acid (H_2_SO_4_, 98%), hydrogen peroxide (H_2_O_2_, 35 wt%), and zinc nitrate hexahydrate (Zn(NO_3_)_2_·6H_2_O, 98%) were obtained from Sigma-Aldrich (St. Louis, MO, USA). Potassium hydroxide (KOH, 30 wt%), N,N-dimethylformamide (DMF, 99%), and acetone (99.5%) were purchased from Samchun Chemicals (Seoul, Republic of Korea).

### 2.2. Preparation of Silicon Template with Inverted Hemisphere Pattern

A positive PR was spin-coated onto a Si (100) wafer at 4000 rpm for 30 s, followed by UV exposure through a chrome mask (Microchemicals Co. (Midland, MI, USA)) using a mask aligner (MA6, Karl Suss; 20 mW). The exposed PR was developed, and reactive ion etching (RIE; Oxford Instrument (Oxford, UK)) was performed using CF_4_ (40 sccm) and O_2_ (5 sccm) gases at 100 W RF power and 10 mTorr to form inverted hemisphere arrays. The remaining PR was removed using acetone, and the Si template was cleaned with piranha solution (1:1 mixture of H_2_SO_4_ and H_2_O_2_)

### 2.3. Fabrication of Hemisphere-Patterned PDMS Template

PDMS was prepared by mixing the base and curing agent in a 9:1 weight ratio. The mixture was poured onto the Si template and cured at 120 °C for 20 min. After curing, the hemisphere-patterned PDMS film (thickness ~400 μm) was peeled off using a razor blade.

### 2.4. Synthesis of ZnO Nanoparticles

ZnO nanoparticles were synthesized via a sol–gel method. Zinc nitrate hexahydrate (7.44 g) was dissolved in 50 mL of distilled water. Separately, 0.58 g of KOH in 15 mL distilled water was prepared and added dropwise to the zinc solution. The mixed solution was stirred vigorously at 80 °C for 1 h. After the reaction, the solution was centrifuged, and the resulting precipitate was dried in a vacuum oven overnight at 70 °C. The dried powder was heat-treated at 300 °C to remove residual impurities.

### 2.5. Electrospinning of PVDF Nanofibers

PVDF (1.7 g) was dissolved in 8.3 g of a solvent mixture of acetone and DMF (volume ratio 7:3) and stirred at 300 rpm at 30 °C for 24 h. The solution was then electrospun onto a roller-type collector rotating at 400 rpm under a voltage of 15 kV, a flow rate of 3.6 mL/h, and a tip-to-collector distance (TCD) of 12 cm.

### 2.6. Electrospinning of ZnO/PVDF Nanofibers

After preparing the PVDF solution as described above, ZnO nanoparticles (0.1 g) were added and stirred at 300 rpm for 30 min, followed by ultrasonication for 1 h. ZnO concentrations were adjusted to 0.6, 0.8, 1.0, 1.2, and 1.4 wt%. The prepared solutions were electrospun using the same conditions described in Section 2.5. To ensure reproducibility, each electrospinning trial was repeated three times under identical conditions, and the average fiber diameters varied within ±5%. Homogeneous ZnO dispersion was confirmed by consistent fiber morphology and SEM imaging across all samples.

### 2.7. Fabrication of ZnO/PVDF/PDMS Nanogenerators

The ZnO/PVDF solution was electrospun directly onto a roller collector attached to a hemisphere-patterned PDMS substrate. Electrospinning was conducted under identical conditions (15 kV, 3.6 mL/h, TCD 12 cm, 400 rpm). After fiber deposition, copper tape and copper wire were connected to the ZnO/PVDF/PDMS composite, which was then encapsulated with polyimide tape to complete the nanogenerator assembly.

### 2.8. Characterization

Morphological analysis was conducted using field-emission scanning electron microscopy (FE-SEM; Carl Zeiss GEMINISEM 300 (Osaka, Japan)). Raman spectra and FT-IR spectra were obtained using a Renishaw inVia Raman microscope and a Bruker VERTEX70, respectively. Crystal structure and phase analyses were performed via X-ray diffraction (XRD; D2 PHASER, Bruker (Billerica, MA, USA)). The relative permittivity was measured using an LCR meter (E4980AL, Keysight (Santa Rosa, CA, USA)). Output voltage measurements were carried out using an oscilloscope (MSO-X 2024A, Keysight).

## 3. Results and Discussion

**Figure 1:**
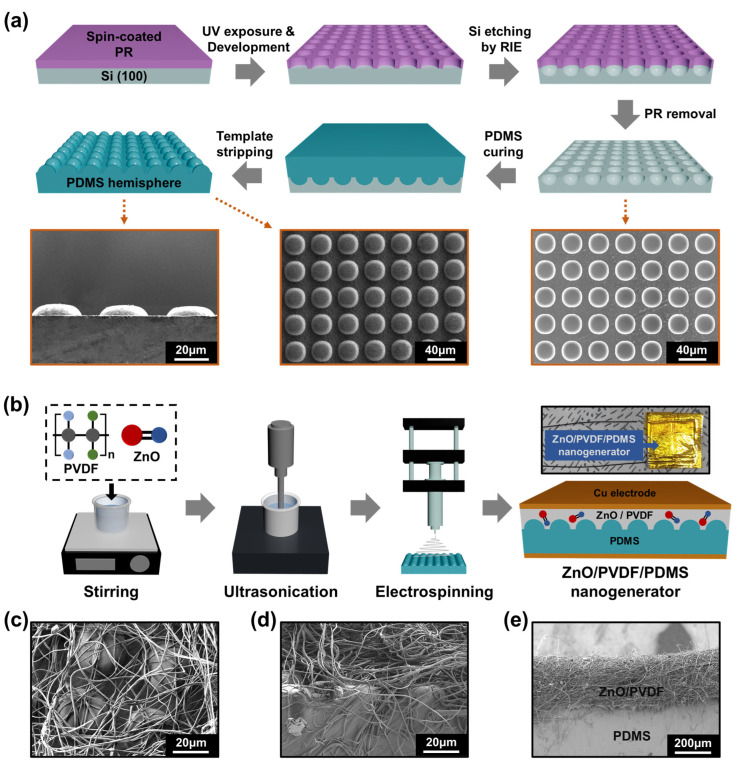
(**a**) Fabrication process of a hemisphere-patterned PDMS template using photolithography and RIE, followed by thermal curing and peeling. SEM images show the inverted hemisphere-patterned Si template and the replicated PDMS hemispheres from side view and top view. (**b**) Schematic of the ZnO/PVDF/PDMS nanogenerator fabrication process, including ZnO/PVDF solution preparation, electrospinning onto PDMS, and integration of Cu electrodes and polyimide insulation. (**c**,**d**) Top-view and side-view SEM images of ZnO/PVDF nanofibers deposited on the hemisphere-patterned PDMS. (**e**) Cross-sectional SEM image of the ZnO/PVDF/PDMS nanogenerator showing a ~300 μm thick nanofiber layer uniformly covering the patterned PDMS surface.

Figure 1 schematically illustrates the complete fabrication process of the ZnO/PVDF/PDMS nanogenerator. The process includes the patterning of an inverted hemispherical Si template, the replication of this structure onto a PDMS substrate, the electrospinning of ZnO/PVDF nanofibers, and the final integration of electrodes and insulation layers to construct the functional device.

The process begins by forming an inverted hemispherical pattern on a Si (100) substrate using standard photolithography and reactive ion etching (RIE), as shown in Figure 1a. A dense array of circular holes, 30 μm in diameter, is formed using a photoresist mask. Plasma etching through RIE generates uniformly arranged inverted hemispheres with a base width of approximately 30 μm and an interspacing of 10 μm, as shown in the bottom-right SEM image in Figure 1a [17]. To replicate this pattern, a PDMS prepolymer is poured onto the etched Si wafer and thermally cured. After curing, the hemisphere-patterned PDMS template is peeled off. The resulting structures demonstrate high morphological fidelity and structural stability. The SEM images shown at the bottom of Figure 1a confirm the successful replication of the hemisphere pattern, where the side-view (left) and top-view (middle) images show PDMS hemispheres with a base diameter of ~30 μm, a height of ~5 μm, and an interspacing of ~10 μm. This corresponds to a surface density of approximately 70,000 hemispheres per cm^2^, indicating a highly ordered microstructure.

Figure 1b illustrates the fabrication of the ZnO/PVDF/PDMS nanogenerator. First, PVDF powder and ZnO nanoparticles are dispersed in a mixed solvent using mechanical stirring followed by ultrasonication. The resulting ZnO/PVDF solution is then electrospun directly onto the hemisphere-patterned PDMS substrate. A roller-type collector enables uniform deposition of the fibers onto the 3D surface. Subsequently, Cu electrodes are attached to the top and bottom of the structure, and polyimide tape is applied to encapsulate the device, preventing unintended charge leakage and ensuring long-term operational stability. Electrospinning not only produces nanofibers but also facilitates β-phase formation in PVDF by inducing both electrical poling and mechanical stretching. The high voltage applied during electrospinning generates an electric field that aligns molecular dipoles, while the rotating collector provides mechanical stretching that further promotes β-phase crystallization [18,19].

Figure 1c,d present top- and side-view SEM images of the electrospun ZnO/PVDF nanofibers deposited on the hemisphere-patterned PDMS. The elastic nature of PDMS enables it to retain its patterned morphology without cracking or deformation after fiber deposition. The nanofibers uniformly fill the interspaces between the hemispheres. Figure 1e shows a cross-sectional FE-SEM image of the ZnO/PVDF/PDMS hybrid structure. The image confirms dense coverage of ZnO/PVDF nanofibers across the curved PDMS surface, with a total thickness of approximately 300 μm. The hemispherical geometry of the PDMS substrate was selected due to its isotropic curvature and mechanical uniformity, which help distribute external stress evenly and reduce localized strain during deformation. Compared to prism or pyramid-shaped patterns that induce a stress concentration at sharp edges, the smooth curvature of hemispheres minimizes mechanical fatigue and enhances durability. Moreover, the curved surface increases the contact area with the electrospun ZnO/PVDF nanofibers, promoting improved interfacial charge transfer. These combined effects contribute to the enhanced and stable piezoelectric output performance observed in the ZnO/PVDF/PDMS nanogenerator. Furthermore, the geometry of the hemisphere-patterned PDMS substrate affects the local deposition thickness of the electrospun ZnO/PVDF nanofibers. Due to their electric field-driven fiber trajectory, nanofibers tend to accumulate more densely in valley regions between hemispheres while convex areas receive less coverage. To minimize thickness variation and ensure a uniform piezoelectric response, the electrospinning duration was optimized to achieve a composite layer thickness of approximately 300 μm, which is consistent with the height scale of the PDMS features (~5 μm).

**Figure 2:**
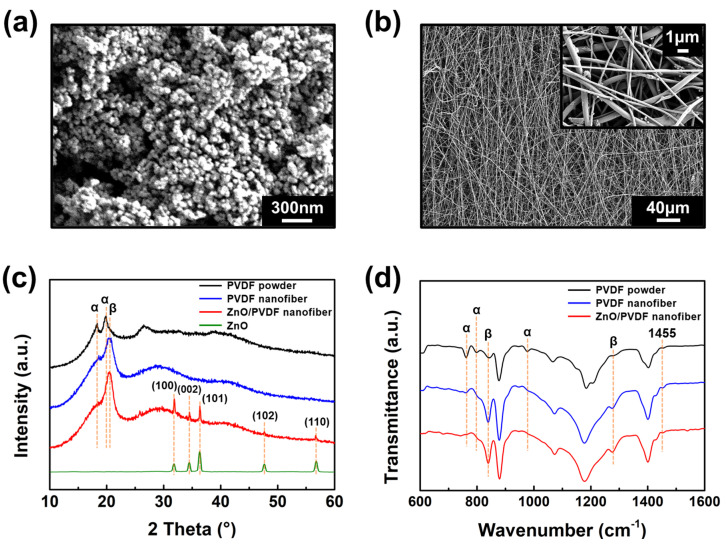
(**a**) FE-SEM image of ZnO nanoparticles synthesized via hydrothermal synthesis. (**b**) FE-SEM image of ZnO/PVDF nanofibers (inset: high-magnification image showing ZnO nanoparticles embedded within PVDF nanofibers). (**c**) XRD spectra of PVDF powder, PVDF nanofibers, ZnO/PVDF nanofibers, and ZnO, showing characteristic α- and β-phase peaks. (**d**) FT-IR spectra of PVDF powder, PVDF nanofibers, and ZnO/PVDF nanofibers, highlighting phase-specific vibrational modes used to calculate the β-phase content.

Figure 2 presents the morphological and structural characterization of the synthesized ZnO nanoparticles and the ZnO/PVDF nanofibers. Figure 2a shows the FE-SEM image of ZnO nanoparticles synthesized via a hydrothermal method, exhibiting a relatively uniform size distribution with diameters ranging from 30 to 50 nm. After calcination, the ZnO nanoparticles retained their spherical morphology and were effectively dispersed into the PVDF matrix via ultrasonication. Figure 2b displays the FE-SEM image of ZnO/PVDF nanofibers fabricated using electrospinning with a rotating drum collector. The fibers are well-aligned with an average diameter of approximately 371 nm, and ZnO nanoparticles are distributed between the fibers. The inset shows a magnified view of the fibers, confirming uniform distribution and separation. The alignment of fibers and the formation of a uniform mat are known to enhance piezoelectric performance by increasing β-phase content [20].

Figure 2c shows the XRD spectra of PVDF powder, PVDF nanofibers, ZnO/PVDF nanofibers, and ZnO powder. The characteristic peaks of PVDF appear at 18.2° and 19.8° (α-phase) and 20.4° (β-phase) [21]. In PVDF powder, the α-phase peaks dominate, whereas both PVDF and ZnO/PVDF nanofibers show reduced α-phase intensity and enhanced β-phase peaks after electrospinning. ZnO exhibits distinct peaks at 31.8° (100), 34.7° (002), 36.4° (101), 47.6° (102), and 56.6° (110), indicating its hexagonal wurtzite structure [22]. These ZnO peaks are also evident in the ZnO/PVDF spectrum, confirming successful incorporation.

Figure 2d presents the FT-IR spectra of PVDF powder, PVDF nanofibers, and ZnO/PVDF nanofibers. The α-phase of PVDF is indicated by peaks at 761, 797, and 975 cm^−1^, while the β-phase is associated with peaks at 839 and 1276 cm^−1^. The peak at 1455 cm^−1^, attributed to CH_2_ bending, appears in both phases [23,24]. PVDF powder shows negligible β-phase content. In contrast, PVDF nanofibers and ZnO/PVDF nanofibers display a marked increase in β-phase peaks and decrease in α-phase peaks after electrospinning due to electric-field-induced dipole alignment. Among these peaks, 761 cm^−1^ (α-phase) and 839 cm^−1^ (β-phase) are commonly used to quantify the β-phase content, denoted as *F(β)* [25]. *F(β)* was calculated using the following Equation [26]:

(1) $$F(\beta )=\;\frac{X_{\beta }}{X_{\alpha }+X_{\beta }}=\;\frac{A_{\beta }}{1.26A_{\alpha }+A_{\beta }\;}$$

where *A_α_* and *A_β_* are the absorbance areas of the α- and β-phase peaks, respectively. The calculated *F(β)* values were 28.7% for PVDF powder, 80.7% for PVDF nanofibers, and 85.6% for ZnO/PVDF nanofibers, indicating a significant enhancement in β-phase crystallinity due to electrospinning and ZnO addition [27]. This β-phase transformation is attributed to electrical poling and mechanical stretching during electrospinning, which align polymer chains and stabilize the piezoelectric β-phase [28,29]. ZnO further promotes β-phase formation by acting as a nucleating agent. Its high dielectric constant intensifies the local electric field at the needle tip during electrospinning, aligning PVDF chains more effectively and accelerating polarization. This synergistic effect results in ZnO/PVDF nanofibers with superior structural and piezoelectric properties compared to pure PVDF [30].

**Figure 3:**
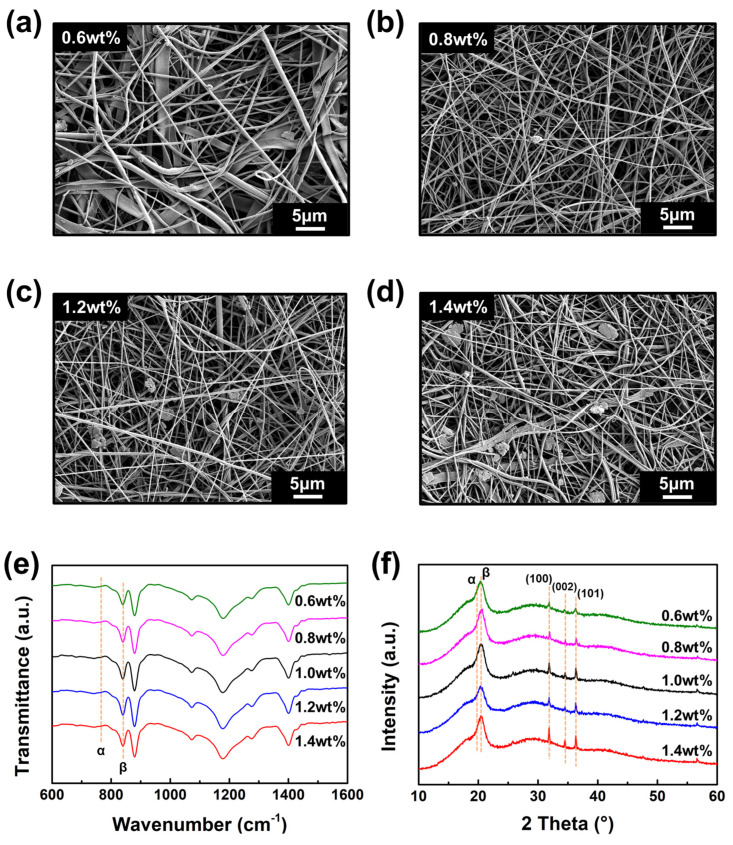
(**a**–**d**) FE-SEM images of ZnO/PVDF nanofibers with different ZnO weight concentrations: (**a**) 0.6 wt%, (**b**) 0.8 wt%, (**c**) 1.2 wt%, and (**d**) 1.4 wt%. (**e**) FT-IR spectra of ZnO/PVDF nanofibers as a function of ZnO content, showing variations in α- and β-phase absorption peaks. (f) XRD spectra of ZnO/PVDF nanofibers with increasing ZnO content, indicating the evolution of ZnO crystallinity.

Figure 3a–d show FE-SEM images of ZnO/PVDF nanofibers with varying ZnO weight concentrations (0.6, 0.8, 1.2, and 1.4 wt%). Fiber diameter plays a key role in determining piezoelectric performance. Thinner fibers enhance β-phase formation, increase the surface area for charge collection, and improve dipole alignment, all of which contribute to higher output voltages [31]. Cylindrical fibers facilitate uniform molecular chain alignment under the influence of electrostatic stretching and rotation, thereby promoting β-phase formation. In contrast, ribbon-like morphologies, which arise from unstable jet dynamics, exhibit lateral spreading and reduced poling efficiency, leading to diminished piezoelectric performance. For both the 0.6 and 1.4 wt% samples, a mixture of cylindrical and ribbon-like morphologies was observed. The ribbon-like fibers tended to exhibit larger diameters, leading to broader diameter distributions: 751 nm for 0.6 wt% and 535 nm for 1.4 wt%. In contrast, the 0.8, 1.0, and 1.2 wt% samples showed more uniform cylindrical morphologies, with narrower diameter distributions and average diameters of 371, 569, and 453 nm, respectively. Among all of the samples, the 0.8 wt% sample had the smallest and most uniform diameter, which is beneficial for achieving stable and enhanced piezoelectric properties [32]. Beyond SEM observations, particle distribution was quantified using ImageJ (version 1.54k) by analyzing grayscale intensity variations across fiber networks. At higher ZnO concentrations (≥1.2 wt%), the increased intensity variation and standard deviation in fiber diameter indicated the onset of nanoparticle agglomeration.

Figure 3e presents the FT-IR spectra of ZnO/PVDF nanofibers with varying ZnO content. As described in Figure 2d, the β-phase fraction *F(β)* was calculated from the absorbance ratio of the α-phase (761 cm^−1^) and β-phase (839 cm^−1^) peaks using Equation (1). The calculated *F(β)* values and relative permittivity for each composition are summarized in Table 1. The pristine PVDF nanofiber without ZnO exhibited a relative permittivity of 14.37, while ZnO addition significantly increased this value due to the high intrinsic permittivity of ZnO. As the average diameter of ZnO/PVDF nanofibers decreased, fiber–fiber interactions intensified, enhancing dipole density [33]. The 0.6 wt% sample, with the largest diameter and lowest ZnO content, showed limited enhancement in *F(β)* and permittivity. Meanwhile, the 0.8, 1.0, and 1.2 wt% samples showed stable electrospinning, finer diameters, and increased *F(β)* values and permittivity due to well-dispersed ZnO nanoparticles. In particular, the 0.8 wt% sample demonstrated the most uniform dispersion of ZnO, which is more effective for improving dielectric behavior than agglomerated ZnO clusters. As a result, this sample exhibited the highest *F(β)* and relative permittivity. Although the 1.4 wt% sample showed slightly lower values, it still ranked second, supported by its higher ZnO content and finer average diameter than the 0.6 wt% sample.

Figure 3f displays the XRD spectra of ZnO/PVDF nanofibers with different ZnO content. With increasing ZnO concentration, the intensity of ZnO diffraction peaks at 31.8° (100), 34.7° (002), and 36.4° (101) becomes more prominent. This trend indicates improved crystallinity and phase purity, as well as greater amounts of wurtzite-phase ZnO. The enhanced diffraction is attributed to the increased number of ZnO nanocrystals embedded within the PVDF matrix [34].

**Figure 4:**
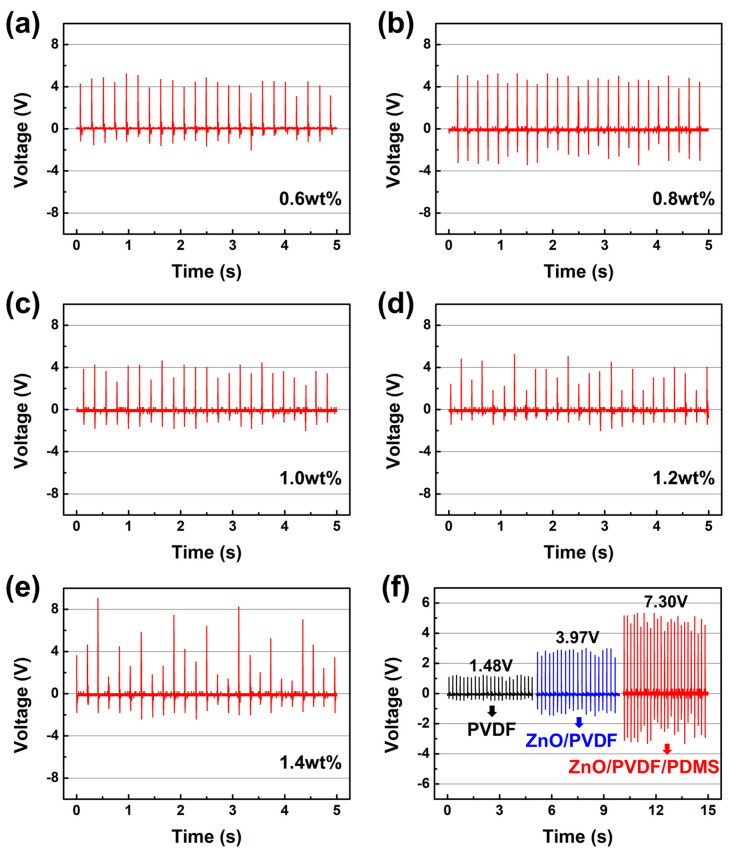
(**a**–**e**) Output voltage profiles of ZnO/PVDF/PDMS nanogenerators with different ZnO weight concentrations under applied forces of approximately 5N: (**a**) 0.6 wt%, (**b**) 0.8 wt%, (**c**) 1.0 wt%, (**d**) 1.2 wt%, and (**e**) 1.4 wt%. (**f**) Comparison of output voltage among pristine PVDF, ZnO/PVDF (0.8 wt%), and ZnO/PVDF/PDMS nanogenerators.

Figure 4a–e show the output voltage profiles of ZnO/PVDF/PDMS nanogenerators fabricated with varying ZnO weight concentrations. The corresponding average voltages and relative standard deviations (RSDs) are summarized in Table 2. Among the samples, the nanogenerator containing 0.8 wt% ZnO exhibited the highest average output voltage (7.30 V) and the lowest RSD (10.8%), indicating both excellent performance and stability. In contrast, nanogenerators with 0.6, 1.0, and 1.2 wt% ZnO exhibited lower average voltages and moderately low RSDs, while the 1.4 wt% sample demonstrated a relatively high average voltage (5.65 V) but suffered from a significantly high RSD (43.4%), implying poor signal consistency that may hinder its application in sensor devices.

These results are attributed to the influence of ZnO content on the electrical and dielectric behavior of the ZnO/PVDF/PDMS composite. At 0.8 wt%, ZnO nanoparticles are optimally dispersed within the PVDF matrix, enhancing field-induced polarization and charge mobility. This leads to improved dipole alignment and greater piezoelectric charge generation, as reflected in the highest *F(β)* value among the samples [35]. On the other hand, the lower ZnO content in the 0.6 wt% sample limits dipole alignment and β-phase formation, resulting in insufficient charge generation despite a relatively low RSD. In the 1.0 wt% sample, reduced *F(β)* due to suboptimal ZnO dispersion further contributes to the decreased average voltage. As ZnO content increases beyond 0.8 wt%, nanoparticle agglomeration begins to occur, especially evident in the 1.2 wt% sample. This agglomeration disrupts homogeneous polarization and charge transport, increasing the RSD and reducing output voltage. Although the 1.4 wt% sample shows a slightly improved average voltage compared to lower concentrations, the severe agglomeration effect leads to highly inconsistent signals. Overall, these findings indicate that 0.8 wt% ZnO is the optimal concentration for balancing charge generation and signal stability.

Figure 4f compares the average output voltage among three nanogenerators: pristine PVDF, ZnO/PVDF (0.8 wt%), and ZnO/PVDF/PDMS (0.8 wt%). The pristine PVDF nanogenerator produced a baseline voltage of 1.48 V. Incorporation of 0.8 wt% ZnO increased the output to 3.97 V, while the addition of a hemisphere-patterned PDMS layer further boosted the output to 7.30 V. These results demonstrate the synergistic effects of ZnO and PDMS in enhancing piezoelectric performance. Compared to recent PVDF-based nanogenerators, our device shows superior performance. For example, a hybrid PVDF/ZrO_2_/ZnO nanocomposite produced 3.2 V under tapping, and a NiO-doped PVDF electrospun fiber generated around 1.5 V [36,37]. Our ZnO/PVDF/PDMS nanogenerator achieved an average output of 7.30 V under ~5 N, indicating a significant enhancement in piezoelectric response. A durability test under 1000 pressing cycles at 5 N revealed that the device retained over 95% of its initial output voltage, confirming the long-term operational stability afforded by polyimide encapsulation.

ZnO nanoparticles, with their high dielectric constant and semiconducting nature, enhance local electric field strength and facilitate better dipole alignment in the PVDF matrix, contributing to improved charge generation [38]. Furthermore, the hemisphere-patterned PDMS plays a critical role in enhancing mechanical stress distribution and strain amplification. The curved surface facilitates more uniform deformation and increases the effective contact area, improving the mechanical interaction between PDMS and the embedded nanofibers [39]. This structure also minimizes stress concentration and mechanical fatigue over repeated cycles, thereby improving long-term performance. Additionally, the microstructured PDMS surface induces localized charge accumulation and promotes dipole orientation in the ZnO/PVDF nanofibers, leading to a more efficient piezoelectric response [40]. The hemisphere pattern also introduces air gaps that cushion external forces, preventing excessive mechanical stress and improving output consistency [41]. The optimized 0.8 wt% ZnO concentration and the incorporation of hemisphere-patterned PDMS together enable the highest and most consistent piezoelectric performance in the ZnO/PVDF-based nanogenerators.

**Figure 5:**
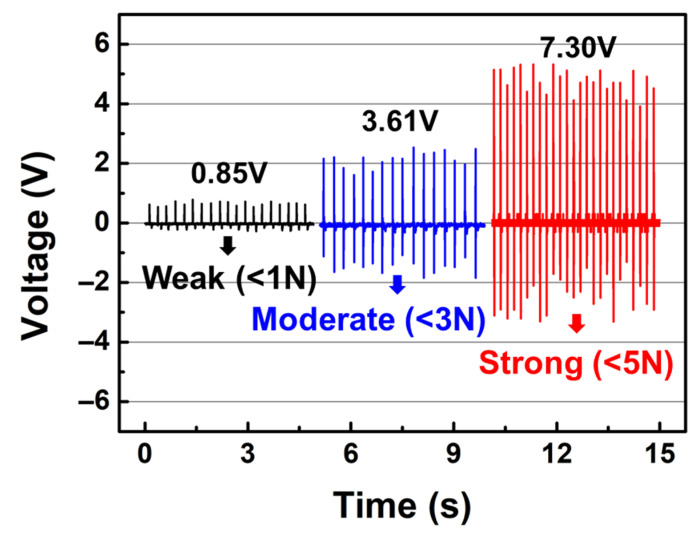
Output voltage responses of ZnO/PVDF/PDMS nanogenerators under applied forces of approximately 1 N, 3 N, and 5 N, demonstrating force-dependent piezoelectric performance.

Figure 5 illustrates the output voltage of ZnO/PVDF/PDMS nanogenerators under varying applied pressures of approximately 1, 3, and 5 N. The corresponding output voltages were measured to be 0.85 V, 3.61 V, and 7.30 V, respectively. The result for the 5 N condition corresponds to the data shown in Figure 4b. Since the contact area during finger-induced pressing is variable and often undefined, the applied mechanical stimulus was quantified in terms of normal force rather than pressure. This approach provides a more practical and consistent metric for evaluating nanogenerator performance under realistic, human-interactive conditions. As expected, higher applied forces result in increased piezoelectric output. The approximate force ranges can be intuitively related to everyday actions: <1 N simulates the pressure of touching a smartphone screen, <3 N mimics pressing a keyboard, and <5 N resembles a light hand clap. These relatable reference points help to contextualize the sensing capability of the nanogenerator under real-life mechanical stimuli.

The improved output with increased applied force is attributed to several factors: (i) stronger mechanical deformation of the piezoelectric layer generates greater electrical charges, (ii) higher forces promote better dipole alignment within the PVDF matrix, (iii) more efficient charge transport and collection are facilitated by enhanced contact and stress distribution, and (iv) the hemisphere-patterned PDMS enhances strain localization and contact area under higher loads. These synergistic effects result in significantly amplified piezoelectric performance with increasing mechanical input [42,43].

## 4. Conclusions

In summary, a hybrid nanogenerator for energy harvesting was fabricated by casting PDMS onto an inverted hemisphere-patterned silicon template, followed by template stripping and electrospinning ZnO-dispersed PVDF nanofibers onto the patterned surface. Among the fabricated devices, the nanogenerator containing 0.8 wt% ZnO exhibited the smallest average fiber diameter (371 nm) with the most uniform diameter distribution. This composition also achieved the highest β-phase fraction (*F(β)*) and a relatively high dielectric constant of 35.8. In terms of device performance, the 0.8 wt% ZnO/PVDF/PDMS nanogenerator delivered the highest output voltage of 7.30 V and showed the most stable and consistent piezoelectric response. Furthermore, the effect of varying applied forces on output performance was investigated, confirming that stronger forces enhance the piezoelectric output. Given its simple and scalable fabrication process, the proposed ZnO/PVDF/PDMS nanogenerator shows great potential for integration into touch-responsive devices, self-powered sensors, and future flexible electronics. These results demonstrate its viability for practical use in wearable systems and energy-harvesting applications.

## Figures and Tables

**Table 1:** *F(β)* and relative permittivity of ZnO/PVDF nanofibers with varying ZnO weight concentrations, measured at 1 kHz.

ZnO Content (wt%)	0.6	0.8	1.0	1.2	1.4
*F(β)* (%)	83.9	85.6	84.2	84.5	85.5
Relative Permittivity (ε_r_)	23.3	35.8	26.7	28.9	35.3

**Table 2:** Average output voltage and relative standard deviation of ZnO/PVDF/PDMS nanogenerators at various ZnO concentrations, evaluated under periodic mechanical stimulation.

ZnO Content (wt%)	0.6	0.8	1.0	1.2	1.4
Average Voltage (V)	5.45	7.30	5.06	4.50	5.65
Relative Standard Deviation (%)	12.2	10.8	14.3	23.3	43.4

## Data Availability

All data are contained within the article.
